# DeSigN: connecting gene expression with therapeutics for drug repurposing and development

**DOI:** 10.1186/s12864-016-3260-7

**Published:** 2017-01-25

**Authors:** Bernard Kok Bang Lee, Kai Hung Tiong, Jit Kang Chang, Chee Sun Liew, Zainal Ariff Abdul Rahman, Aik Choon Tan, Tsung Fei Khang, Sok Ching Cheong

**Affiliations:** 1 Department of Oral & Maxillofacial Clinical Sciences, Faculty of Dentistry, University of Malaya, 50603 Kuala Lumpur, Malaysia; 2 Oral Cancer Research Group, Cancer Research Malaysia, No. 1, Jalan SS12/1A, 47500 Subang Jaya, Selangor Malaysia; 3 Data Intensive Computing Centre, Research Management & Innovation Complex, University of Malaya, 50603 Kuala Lumpur, Malaysia; 4 Department of Computer System & Technology, Faculty of Computer Science & Information Technology, University of Malaya, 50603 Kuala Lumpur, Malaysia; 5 Centre for Data Science, University of Malaya, 50603 Kuala Lumpur, Malaysia; 6 Division of Medical Oncology, School of Medicine, University of Colorado Anschutz Medical Campus, Aurora, CO 80045 USA; 7 Institute of Mathematical Sciences, University of Malaya, 50603 Kuala Lumpur, Malaysia

**Keywords:** Cell line, Gene expression, DeSigN, Cancer, Drug repurposing

## Abstract

**Background:**

The drug discovery and development pipeline is a long and arduous process that inevitably hampers rapid drug development. Therefore, strategies to improve the efficiency of drug development are urgently needed to enable effective drugs to enter the clinic. Precision medicine has demonstrated that genetic features of cancer cells can be used for predicting drug response, and emerging evidence suggest that gene-drug connections could be predicted more accurately by exploring the cumulative effects of many genes simultaneously.

**Results:**

We developed DeSigN, a web-based tool for predicting drug efficacy against cancer cell lines using gene expression patterns. The algorithm correlates phenotype-specific gene signatures derived from differentially expressed genes with pre-defined gene expression profiles associated with drug response data (IC_50_) from 140 drugs. DeSigN successfully predicted the right drug sensitivity outcome in four published GEO studies. Additionally, it predicted bosutinib, a Src/Abl kinase inhibitor, as a sensitive inhibitor for oral squamous cell carcinoma (OSCC) cell lines. In vitro validation of bosutinib in OSCC cell lines demonstrated that indeed, these cell lines were sensitive to bosutinib with IC_50_ of 0.8–1.2 μM. As further confirmation, we demonstrated experimentally that bosutinib has anti-proliferative activity in OSCC cell lines, demonstrating that DeSigN was able to robustly predict drug that could be beneficial for tumour control.

**Conclusions:**

DeSigN is a robust method that is useful for the identification of candidate drugs using an input gene signature obtained from gene expression analysis. This user-friendly platform could be used to identify drugs with unanticipated efficacy against cancer cell lines of interest, and therefore could be used for the repurposing of drugs, thus improving the efficiency of drug development.

**Electronic supplementary material:**

The online version of this article (doi:10.1186/s12864-016-3260-7) contains supplementary material, which is available to authorized users.

## Background

The drug discovery and development pipeline is a long and arduous process, one that is resource-intensive and time-consuming, making these the main barriers for rapid drug development. Furthermore, the attrition rate is high, underscoring the need to improve strategies in drug development and in expanding the usage of already approved drugs [1]. Fortunately, the availability of a large pool of drugs provides convenient candidates for drug repurposing, which can contribute to reducing the time for finding new, effective chemotherapeutic strategies [2]. The current challenge is to develop discovery pipelines to prioritize testing of already approved drugs, particularly in cancers with limited chemotherapy options, such as oral cancer [3]. Lessons from laboratory and clinical studies have demonstrated that genetic features of tumours either in the form of mutational data or gene expression signatures could be used to predict response to targeted therapies, and this has formed the basis of precision medicine that is currently practised in the clinic [4–6]. To extend on the advancements in our ability to characterize the cancer genome to unprecedented depth, these information can be used to link genetic features to drug response, which affords an opportunity to systematize the testing of drug candidates for expanding the spectrum of available cancer drugs for treatment.

Since the late 1980s, the NCI-60 panel of cancer cell lines has been used to systematically identify anti-cancer compounds and more recently, to identify biomarkers of response [7, 8]. In 2012, the repertoire of cancer cell lines used was expanded substantially with the inclusion of new data from the Genomics of Drug Sensitivity in Cancer (GDSC) and Cancer Cell Line Encyclopedia (CCLE) projects where 707 and 860 cancer cell lines respectively were assembled for anti-cancer drug testing. Uniquely, more than 13 cancer types are represented in these panels, and more importantly, these cell lines are well-characterised with respect to their gene expression and mutational information [9, 10]. Additionally, more than 50% of these cell lines were subjected to high-throughput drug screening and their response to a large panel of drugs have been documented systematically [9, 10].

The development of computational tools that could take advantage of the availability of high throughput gene expression data to mine patterns of association between drug sensitivity and gene expression signatures began with the seminal work by Lamb et al. who developed the Connectivity Map (CMap) algorithm [11]. Subsequently, other bioinformatics tools were developed. For example, NFFinder searches for relationships between drugs, diseases and a phenotype of interest using transcriptomic data as input [12]. Using the same concept, the drug-to-protein associations were evaluated by the DMAP tool that resulted in the formation of 438,004 drug-to-protein effect relationships [13]. The Functional Module Connectivity Map (FMCM), which extends CMap by constructing a functional network of a set of differentially expressed genes, showed validation results for four drugs that could affect cell viability in colorectal cancer cell lines [14].

While GDSC provides large amounts of drug response data from arrays of cell lines, additional analyses are needed to extrapolate drug efficacy to new datasets. For example, GDSC shows that the head and neck cancer cell lines FADU and HSC-3 are reported to respond to the heat shock protein 90 (Hsp90) inhibitor 17-AAG [9]. However, predicting which inhibitors are likely to be efficacious in new cell lines derived from cancer patients remains a challenge.

To exploit the GDSC data for predicting drug sensitivity, we developed DeSigN (Differentially Expressed Gene Signatures - Inhibitors), a CMap-inspired [11] bioinformatics pipeline that enables gene expression patterns from experimental data to be linked to gene expression patterns associated with drug response in a cancer cell line database. To demonstrate proof-of-concept of the practical usefulness of DeSigN, we conducted two validation experiments. The first involves the examination of reported efficacy of drug candidates against four different cancer cell lines that are prioritized by DeSigN. The second is an experimental validation of the sensitivity of a set of oral squamous cell carcinoma (OSCC) cell lines to bosutinib, a Src/Abl kinase inhibitor that is currently used for treating leukemia but predicted by DeSigN to be effective against OSCC cell lines.

## Methods

### Differentially Expressed Gene Signatures - Inhibitors (DeSigN) platform

**Fig. 1:**
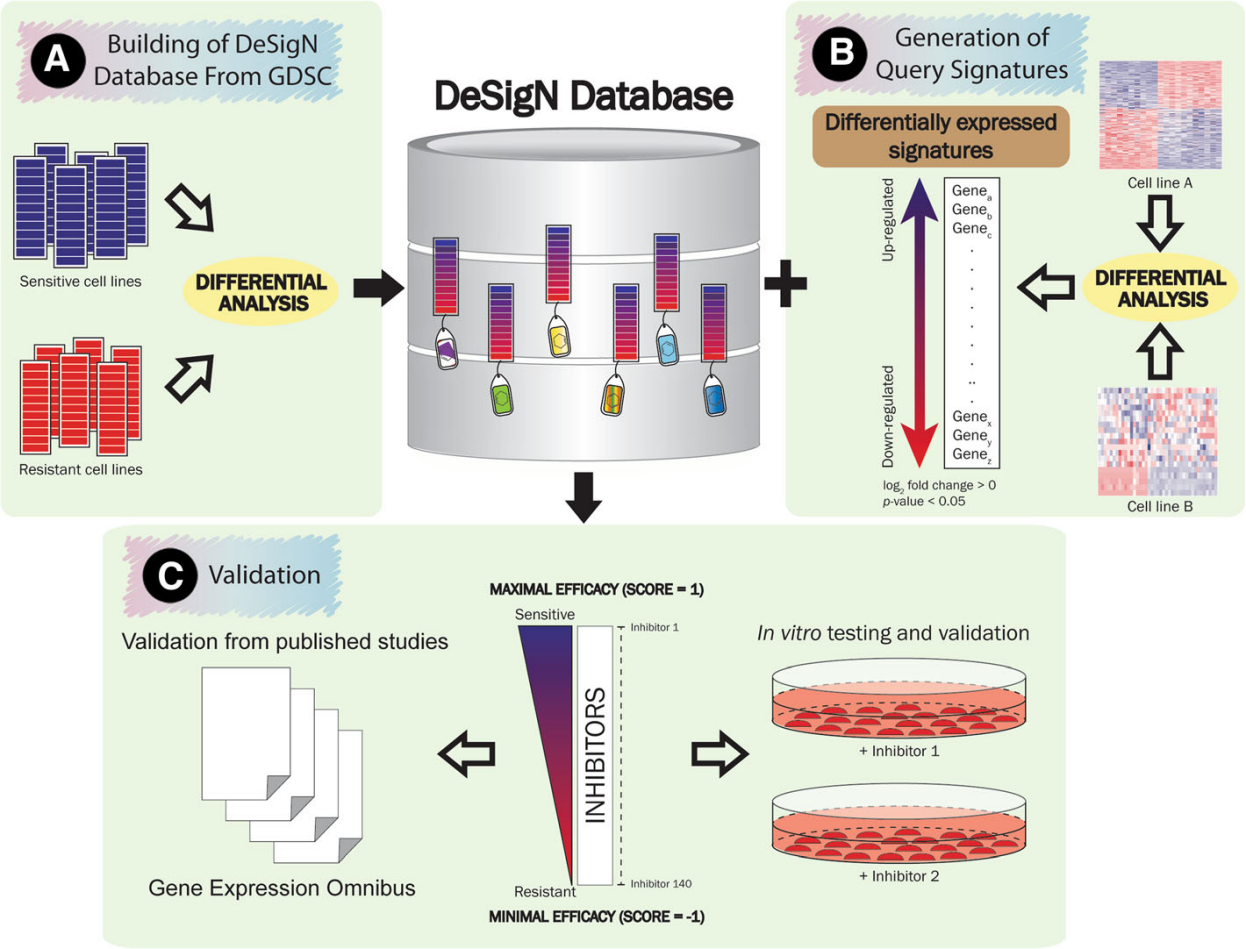
Workflow of DeSigN. **a** A reference database of cell lines that are sensitive and resistant to drugs available in the GDSC database was created. Version 1.0 contains 140 drugs with their unique ranked-based gene signatures. **b** Differential expressed gene signatures are generated from differential expression analysis of cell lines from two distinct experimental conditions, e.g. cell line gene expression data from tumour samples versus normal samples. The up and down-regulated genes (log_2_ fold change > 1 and *p-*value < 0.01) thus selected will be used to query the DeSigN database. **c** A ranked-based list of inhibitors is generated, with Connectivity Score between 1 (maximal efficacy) and −1 (minimal efficacy). This allows users to prioritize the testing of these candidates

DeSigN is a web-based bioinformatics tool for associating gene signatures with drug response phenotype based on IC_50_ data, with the aim of identifying novel drugs that have good potential to be repurposed for cancer therapy. The DeSigN algorithm (Fig. 1) consists of three key components: (i) a reference database that contains a set of pre-defined gene expression profiles associated with drug response data to 140 drugs; (ii) a set of differentially expressed gene (DEG) signatures as query input and (iii) a pattern-matching algorithm for evaluating similarity between the query gene signature and drug-associated gene expression profiles in the reference database.

#### Reference database

**Fig. 2:**
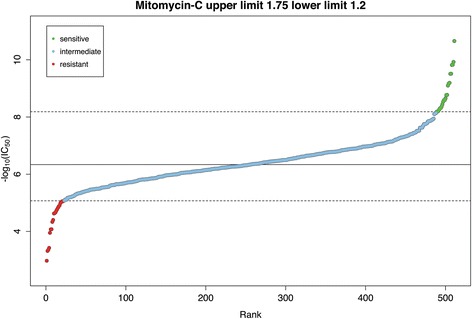
Example of –log_10_(IC_50_) rank plot to define drug response phenotype. The solid line represents the median IC_50_ values of inhibitor Mitomycin-C whereas the lower and upper dashed lines represent the cut-off for classifying cell lines into sensitive or resistant phenotypes, respectively

We built the reference database using baseline microarray data and drug sensitivity data obtained from the Genomics of Drug Sensitivity in Cancer (GDSC) project. We first downloaded the raw CEL microarray data files of solid tumour cell lines from GDSC [9] (normalized using the MAS5 algorithm). The probe sets were collapsed to gene symbols using Gene Set Enrichment Analysis [15] with HT HG-U133A chip as reference, this process resulted in 12,772 unique genes. For each drug, we classified the cancer cell lines’ drug response phenotype (resistant or sensitive) in the following way. We first ranked the cell lines by their IC_50_ values (lowest to highest). Cell lines with IC_50_ that were *U* standard deviations larger than the median IC_50_ of all cell lines were considered to be resistant; those that were *L* standard deviations smaller were considered to be sensitive. We chose the parameters *U* and *L* carefully on a case-by-case basis. These two cut-offs were generally values where sharp transitions in IC_50_ were observed in the scatter plot of –log_10_(IC_50_) against rank. About 20 cell lines each from the sensitive and resistant phenotype were thus defined. The list of sensitive and resistant cell lines defined for the 140 inhibitors in DeSigN is provided in Additional file 1: Table S1. An example for the drug Mitomycin-C is shown in Fig. 2.

Differential expression of microarray gene expression data for the sensitive and the resistant phenotype was done using the Linear Models for Microarray data (limma) algorithm [16]. The result from limma for each inhibitor was sorted and converted into ranked lists according to the gene’s moderated *t*-statistic (rank 1 for largest value). This reference database was used to connect the queries and return rank-ordered list of inhibitors for a particular query (Fig. 1a).

#### Query signature

Differentially expressed genes (DEG) obtained from microarray or RNA-Seq gene expression data of cell lines of two different phenotype classes were used to query DeSigN. DEGs were selected using joint filtering of *p-*value and fold change [17], with threshold value set at log_2_ fold change > 1 and *p-*value < 0.01 (Fig. 1b).

#### Pattern-matching algorithm

A pattern-matching algorithm based on the nonparametric Kolmogorov-Smirnov (KS) statistic [11] was used to associate query signatures to the drug-specific, rank-ordered gene expression profile database. The KS test is a rank-based pattern matching approach implemented in the Connectivity Map [11], and its goal is to correlate inhibitors in GDSC that enrich for similar DEG based on the IC_50_ drug sensitivity profiles.

The Connectivity Score is computed according to [11] as follows. Let *N* be the total number of genes in the reference database, and *T* the number of genes in the query signature for up- or down-regulated genes. For every drug in the reference database, we compute the rank-ordered (using moderated *t*-statistic) list *R* for all *N* genes. Let *j* index the query genes in such a way that *R*(*j*), the rank of the *j*-th gene in the *N* total number of genes, is monotone increasing. For *j* = 1, 2, …, *T,* we compute the following two values for each up- and down-regulated gene signatures:

$$ a=\underset{1\le j\le T}{ \max}\left\{\frac{j}{T}-\frac{R(j)}{N}\right\}; $$

$$ b=\underset{1\le j\le T}{ \max}\left\{\frac{R(j)}{N}-\frac{\left(j-1\right)}{T}\right\}. $$

Subsequently, for each inhibitor *i*, the KS-like statistics for up- and down-regulated query gene signature, *ks*_*up*_^*i*^ and *ks*_*down*_^*i*^, are computed as (subscript omitted)

$$ k{s}^i=\left\{\begin{array}{c}\hfill a,\  if\ a>b;\hfill \\ {}\hfill 0,\  if\ a=b;\hfill \\ {}\hfill -b,\  if\ a<b.\hfill \end{array}\right. $$

The Enrichment Score (*ES* ^*1*^) for drug *i* in the reference database is set to zero if both *ks*_*up*_^*i*^ and *ks*_*down*_^*i*^ have the same sign; otherwise, *ES* ^*i*^ = *ks*_*up*_^*i*^ − *ks*_*down*_^*i*^. The Connectivity Score (*S* ^*i*^) for non-zero instances is a normalized Enrichment Score computed as:

$$ {S}^i=\left\{\begin{array}{c}\hfill \frac{E{S}^i}{P},\  if\ E{S}^i>0;\hfill \\ {}\hfill -\left(\frac{E{S}^i}{Q}\right),\  if\ E{S}^i<0,\hfill \end{array}\right. $$

where *P* = max_*i*_ *ES* ^*i*^ and *Q* = min_*i*_ *ES* ^*i*^ are the normalizing constants.

DeSigN returns a ranked list of inhibitors that have the highest Connectivity Score between the DEG and the ranked-order gene expression profiles in the reference database, with *S* ranging between 1 (maximal efficacy) and −1 (minimal efficacy) (Fig. 1c).

To evaluate the statistical significance of *S* ^*i*^, we used a permutation approach to simulate the null distribution of *S* ^*i*^. Thus, *m* random gene sets, each having the same size as the size of the input gene signature, were simulated. Each gene set then yields *S*_*random*_^*i*^(*k*), where *k* indexes the random gene set. The *p-*value was computed as

$$ p- value = \left\{\begin{array}{c}\hfill \frac{1}{m}{\displaystyle \sum_{k=1}^m}{I}_{\left({S}_{random}^i(k) > {S}^i\right)},\kern0.5em  if\ {S}^i>0;\hfill \\ {}\hfill \frac{1}{m}{\displaystyle \sum_{k=1}^m}{I}_{\left({S}_{random}^i(k) < {S}^i\right)},\kern0.5em  if\ {S}^i<0;\hfill \\ {}\hfill max\left\{\frac{1}{m}{\displaystyle \sum_{k=1}^m}{I}_{\left({S}_{random}^i(k) > {S}^i\right)}\right.,\left.\frac{1}{m}{\displaystyle \sum_{k=1}^m}{I}_{\left({S}_{random}^i(k) < {S}^i\right)}\right\},\kern0.5em  if\ {S}^i=0,\hfill \end{array}\right. $$

where *I*_*A*_ is the indicator function that takes the value 1 if event A occurs, and 0 otherwise. Here, we set *m* = 1000.

### The DeSigN web interface

The DeSigN website is freely available at http://design.cancerresearch.my/. Its web interface is implemented in PHP (v7.0) with the support of jQuery (v1.4.2), and hosted using the Apache Server. The reference database is generated and managed using MySQL database (v5.5.49). DeSigN makes use of the AJAX feature to quickly load content without reloading the pages. All queries are sent to the Java-based computing cluster to perform parallel computation. A help document providing a guide for users to query and navigate DeSigN is available in the website, with examples given. Except the pattern-matching algorithm, which was programmed in Java and the Graphical User Interface (GUI), which was built using PHP, the other methods were implemented in R version 3.3.0.

### NCBI Gene Expression Omnibus (GEO) datasets

To demonstrate how DeSigN could be used to predict candidate drugs, we used differentially expressed genes generated from ER-positive breast cancer versus normal tissue reported by Clarke et al. [18] that can also be accessed from the NCBI Gene Expression Omnibus (GEO) database under the accession number GSE42568. In addition, four drug sensitivity studies published in the NCBI GEO database were used to validate DeSigN (Table 1). The microarray gene expression data from these five GEO studies were subjected to differential analysis using the GEO2R function provided by NCBI (version info: R 2.14.1, Biobase 2.15.3, GEOquery 2.23.2, limma 3.10.1). For the four validation sets, we defined sensitive cell lines as having IC_50_ < 1 μM and resistant cell lines as having IC_50_ > 1 μM. The choice of these four studies was guided by several inclusion and exclusion criteria. We included studies where: (i) The median of the distribution of gene expression values of each sample were more or less equal; (ii) The subject of the drug sensitivity study was *Homo sapiens*; (iii) Drug treatment was given for at least 48 h; (iv) Only one inhibitor was used. We excluded blood cancer-related studies. For each study, a list of DEG was identified and used to query DeSigN.

**Table 1:** GEO studies used to validate DeSigN prediction

GEO reference	Drug	Response	Number of sensitive samples	Number of resistant samples	Platform	Reference
GSE4342	Gefitinib	Sensitive	17	12	GPL96	Coldren et al. [24]
GSE16179	Lapatinib	Sensitive	3	3	GPL570	Liu et al. [35]
GSE9633	Dasatinib	Sensitive	11	5	GPL571	Wang et al. [36]
GSE35141	Gemcitabine	Resistant	6	6	GPL4133	Saiki et al. [37]

### Cell culture

Five oral squamous cell carcinoma (OSCC; ORL-48, ORL-150, ORL-156, ORL-196 and ORL-204) and three normal oral keratinocyte (NOK) cultures previously developed in our laboratory [19] were used to validate bosutinib, a drug candidate predicted by DeSigN to be effective. The RNA-Seq data of these cells were subjected to differential analysis (OSCC versus NOK) using DESeq2 [19, 20]. DEG generated from DESeq2 was used as the query signature in DeSigN to shortlist candidate drugs for experimental validation.

All ORL cell lines and HSC-4 (sensitive control for response to bosutinib) were cultured in Dulbecco’s Modified Eagle Medium (DMEM)/F12 (1:1) supplemented with 10% (v/v) heat-inactivated fetal calf serum (FBS), 100 IU Penicillin/Streptomycin and 0.5 μg/ml hydrocortisone as described previously [19]. NOK were cultured in keratinocyte serum-free media (KSFM; GIBCO, Carlsbad, CA, USA) supplemented with 25 μg/ml bovine pituitary extract, 0.2 ng/ml epidermal growth factor, 0.031 mM calcium chloride and 100 IU Penicillin/Streptomycin (GIBCO, Carlsbad, CA, USA) as described previously [19]. The breast cancer cell line MCF7 (resistant control for response to bosutinib) was cultured in RPMI 1640 medium (GIBCO, Carlsbad, CA, USA) supplemented with 10% (v/v) heat-inactivated FBS and 100 IU Penicillin/Streptomycin. All cultures were incubated in a humidified atmosphere of 5% CO_2_ at 37 °C.

### Viability assay using 3-(4,5-dimethylthiazol-2-yl)-2,5-diphenyltetrazolium bromide (MTT)

The effect of bosutinib on the selected OSCC cell lines was determined using MTT assay with 1.5–8 × 10^3^ cells per well as described previously [19]. Cells were treated with 0.04–5 μM of bosutinib, and cell viability was measured after 72 h of treatment. DMSO (0.5%) served as vehicle control. The two-sample *t*-test was used to assess whether the difference in the sample mean of IC_50_ between the tested cell lines was statistically significant (*p*-value < 0.05). Experiments were repeated at least three times.

### Apoptosis assay

Apoptosis was quantified using a FITC Annexin V Apoptosis Detection Kit (BD Biosciences, San Jose, CA, USA) according to the manufacturer’s instructions. Briefly, floating and attached cells were collected at 24, 48 and 72 h after bosutinib treatment at 1 μM, and then stained using FITC Annexin V/Propidium iodide (PI). Apoptosis detection was performed using BD FACSCANTO™ II flow cytometer and data was analyzed using the BD FACSDiva™ software (BD Biosciences, San Jose, CA, USA). For each of the three time points, the two-sample *t*-test was used to test whether the mean of total number of apoptotic events differed significantly (*p*-value < 0.05) between bosutinib-treated cells and the vehicle control (0.01% DMSO) cells. Experiments were repeated at least two times.

### Proliferation assay

The anti-proliferative effect of bosutinib on the OSCC cell lines were examined using Click-iT EdU Cell Proliferation Assay Kit (Invitrogen, Carlsbad, CA, USA) as previously described [19]. The cell lines ORL-48, ORL-204 and ORL-196 were treated with 0.3–3 μM bosutinib, for 24 h and cell proliferation evaluation was based on 5-ethynyl-2′-deoxyuridine (EdU) incorporation according to the manufacturer’s protocol. Images were captured from 4 to 11 different fields of each treatment concentration and further analyzed using EBImage [21]. The percentage of EdU-labelled cells was expressed as the percentage of red fluorescent nuclei over the total number cells reflected by DAPI-stained nuclei and the data is presented as relative percentage compared to control cells (0 μM). The two-sample *t*-test was used to test whether the difference in the relative percentage of EdU^+^ cells differed significantly (*p*-value < 0.05) between treatment and vehicle control for the three cell lines. Experiments were repeated at least two times.

## Results

### Running DeSigN

**Fig. 3:**
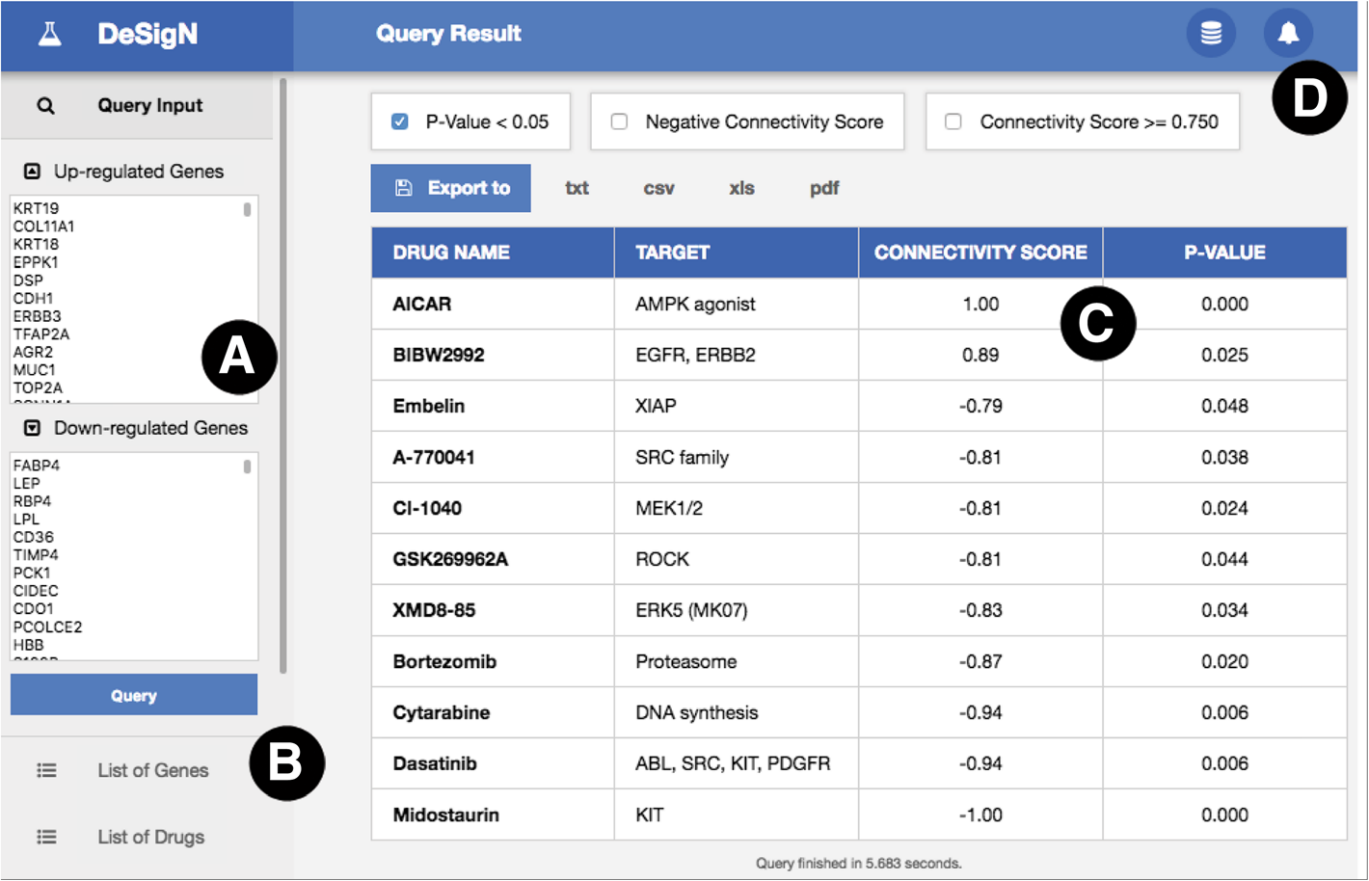
Example of a result page from DeSigN. Users can supply the differentially expressed genes for their study in the boxes in the Panel (**a**). Additional information such as list of genes and drugs currently available in DeSigN can be found in Panel (**b**). Panel (**c**) shows the Connectivity Score results. Error messages (e.g. invalid gene symbols or redundant gene symbol) are produced in Panel (**d**) to alert users of potential problems with input data

To demonstrate how DeSigN can be used to generate a list of prioritized candidate drugs, we tested differentially expressed genes (DEG) generated from ER-positive breast cancer cell line compared to normal tissues (GSE42568; Fig. 3a) [18]. From the database (Fig. 3b), DeSigN returned a list of 11 ranked inhibitors together with their target proteins (Fig. 3c). Of note, the two top-scoring drugs, AICAR and BIBW2992 are drugs that are actively being studied as therapeutics against ER-positive breast cancer. The drug AICAR, which targets AMPK, have shown to have anti-proliferative effects in ER-positive breast cancer cell lines [22]. Further, a Phase II clinical trial demonstrated that BIBW2992 was able to induce stable disease in more than 50% of ER-positive metastatic breast cancer that has progressed on letrozole monotherapy when used in combination with letrozole [23]. DeSigN also predicted resistance of ER-positive breast cancer cells against drugs with strong negative Connectivity Score such as dasatinib and midostaurin. The list of DEG from GSE42568 used to query DeSigN is provided in Additional file 2: Table S2.

### Validation results

**Fig. 4:**
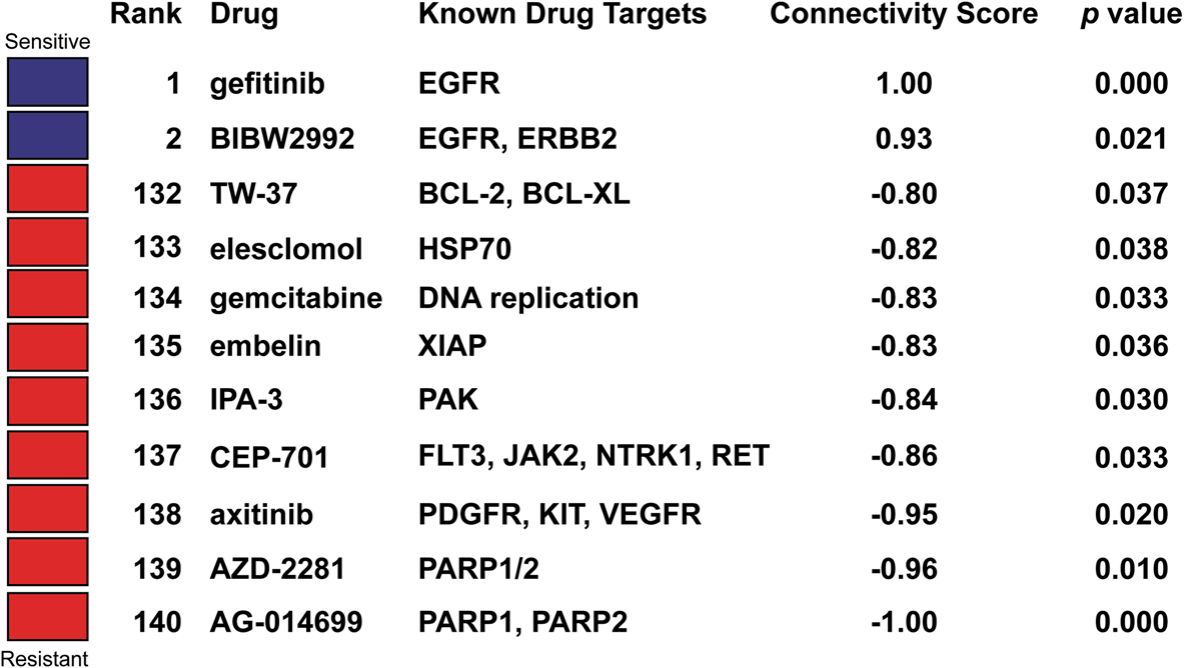
DeSigN prediction result for GSE4342. Gefitinib is predicted to be sensitive, with significant Connectivity Score of 1.00 and *p-*value < 0.001

GSE4342 is a study that demonstrated the sensitive response of 17 non-small cell lung cancer (NSCLC) cell lines to gefitinib (EGFR-inhibitor) treatment [24]. By querying DeSigN using 205 up- and 137 down-regulated genes, two drugs - gefitinib and BIBW2992, were returned with positive Connectivity Score (*p-*value < 0.05). As expected, gefitinib was returned as the top-ranked inhibitor with Connectivity Score of 1.00 and *p*-value < 0.001 (Fig. 4). Interestingly, BIBW2992, also known as afatinib, a second generation EGFR inhibitor, is ranked second with a significant Connectivity Score of 0.93 (*p-*value = 0.021).

For each of the four studies, DeSigN returned Connectivity Scores that correctly correlated drug response outcome that was consistent with the respective published GEO studies. In all these studies, DeSigN successfully associated input gene signatures with the right drugs, all with statistically significant *p*-values (Table 2). The list of DEG of each study used to query DeSigN is provided in Additional file 3: Table S3; Additional file 4: Table S4; Additional file 5: Table S5 and Additional file 6: Table S6.

**Table 2:** NCBI GEO datasets validation summary

GEO reference	Reported drug	Expected drug sensitivity	DeSigN rank	DeSigN drug	Target	Connectivity Score	*p*-value
GSE4342	Gefitinib	Sensitive	1	Gefitinib	EGFR	1.00	0.000
GSE16179	Lapatinib	Sensitive	6	Lapatinib	EGFR, ERBB2	0.87	0.015
GSE9633	Dasatinib	Sensitive	6	Dasatinib	ABL, SRC, KIT, PDGFR	0.83	0.025
GSE35141	Gemcitabine	Resistant	129	Gemcitabine	DNA replication	−0.83	0.025

### Using DeSigN to shortlist potentially efficacious inhibitors for OSCC cell lines

**Fig. 5:**
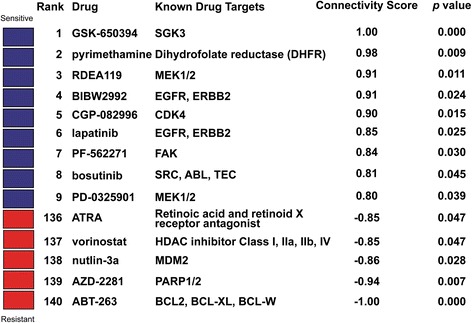
DeSigN prediction results for OSCC cell lines. Nine drugs were predicted to be efficacious (*blue box*) whereas five were predicted to have minimal efficacy on the OSCC cell lines (*red box*)

As we demonstrated that DeSigN could correctly predict drug response from published data, we next used DeSigN to identify inhibitors that could control the growth of OSCC cell lines. The gene signature for differential gene expression between OSCC cell lines and NOK contained 69 and 86 up- and down-regulated genes (Additional file 7: Table S7). Nine potentially efficacious drugs were returned by DeSigN, with another five drugs were predicted to be resistant (Fig. 5), with *p-*values < 0.05. The ranking results corroborated well with recent findings. Two of the candidates, BIBW2992 (ranked fourth) and bosutinib (ranked eighth), have been recently reported to be effective against head and neck squamous cell carcinoma (HNSCC) cell lines [25]. We set out to further evaluate the efficacy of bosutinib, which targets Src and Abl, as it is a recently FDA-approved drug for treating BCR-ABL leukemic patients and have no known effects against HNSCC or OSCC, therefore the efficacy of bosutinib is unanticipated when used against OSCC cell lines.

**Fig. 6:**
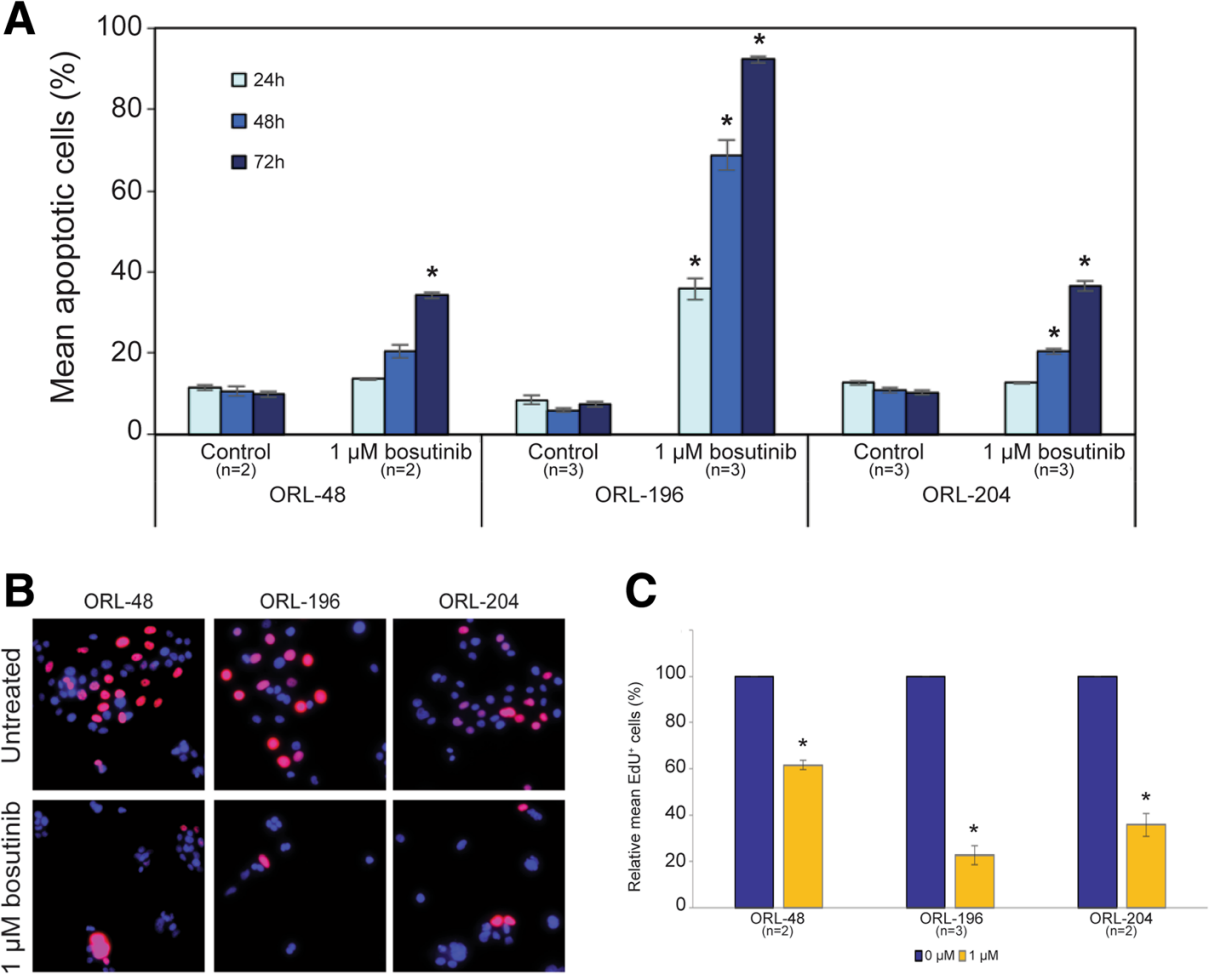
Differential sensitivity of OSCC cell lines, ORL-48, ORL-196 and ORL-204 to bosutinib. **a** Bosutinib induced apoptosis in OSCC cell lines. ORL-48, ORL-196 and ORL-204 cells were treated with 1 μM of bosutinib for 24, 48 and 72 h followed by Annexin V/PI staining coupled with flow cytometry analysis. The bars represent mean percentage of apoptotic cells ± SE of each cell line of at least two experiments. * denotes *p*-value < 0.05 relative to control cells. **b** Bosutinib inhibited the proliferation of OSCC cells as demonstrated by the reduced number of proliferating cells (*red stained cells*) following 72 h treatment at 1 μM. The *blue*-*stained* nuclei represent the total number of cells in a field while the *red*-*stained* nuclei represent proliferating cells that have incorporated the EdU label. **c** OSCC cell proliferation was significantly inhibited by bosutinib with ORL-196 showing the greatest sensitivity (~80% inhibition) followed by ORL-204 (~70% inhibition) and ORL-48 (~50% inhibition) after bosutinib treatment at 1 μM for 72 h. * denotes significance of *p*-value < 0.05

For experimental validation of bosutinib’s efficacy against OSCC, we tested it in three OSCC cell lines (ORL-196, ORL-204 and ORL-48). All three OSCC cell lines (Table 3, Additional file 8: Figure S8) were found to have significantly lower mean IC_50_ value compared to their sensitive head and neck squamous cell carcinoma control (HSC-4, IC_50_: 1.82 μM). Against the resistant control, MCF-7, all three OSCC cell lines also had significant lower mean IC_50_ (Table 3, Additional file 8: Figure S8). This finding is supported by fluorescence-activated cell sorting (FACS) analysis of the cells where bosutinib induced cell death in OSCC cell lines in a time-dependent manner (Fig. 6a, Additional file 9: Table S9). In particular, ORL-196 cells were found to be more responsive to bosutinib, as close to 35% of apoptotic cells were detected as early as 24 h of treatment, while ORL-48 and ORL-204 remained unaffected. By 72 h, a significant number of apoptotic cells (35–90%) were detected in all the OSCC cell lines (*p*-values < 0.01), indicating the cytotoxic effect of bosutinib in these OSCC cells.

**Table 3:** Mean IC_50_ relative to HSC-4 and MCF7 (μM)

OSCC Cell lines	Mean IC_50_ ± SE	-log_10_(*p-*value) relative to HSC-4	-log_10_(*p-*value) relative to MCF7
ORL-196 (*n* = 4)	0.75 ± 0.03	5.8	1.9
ORL-204 (*n* = 3)	0.90 ± 0.04	3.6	1.9
ORL-48 (*n* = 5)	1.19 ± 0.05	4.1	1.9
HSC-4 (*n* = 3)	1.82 ± 0.03	-	-
MCF7 (*n* = 3)	12.22 ± 1.32	-	-

Further confirmation from the Click-iT EdU cell proliferation assay showed clearly that bosutinib inhibited the proliferation of ORL-48, ORL-196 and ORL-204 cells as demonstrated by the significant reduction in the number of proliferating cells (red-stained cells) compared to the non-treated cells (Fig. 6b). ORL-196 and ORL-204 demonstrated growth inhibition of ~70–80% (*p*-value = 0.03, *n* = 3; *p*-value = 0.049, *n* = 2 respectively) whilst ORL-48 showed growth inhibition of ~40% following bosutinib treatment at 1 μM for 72 h (*p*-value = 0.04, *n* = 2) (Fig. 6c, Additional file 10: Table S10 and Additional file 11: Figure S11). The level of inhibition in the OSCC cell lines corroborated well with their mean IC_50_ value for bosutinib. Taken together, these biological observations demonstrated that bosutinib confers anti-proliferative and cytotoxic effects in the tested OSCC cell lines.

## Discussion

We have developed DeSigN, a web-based bioinformatics tool that allows users to query large public database of cancer cell line gene expression and drug response data such as GDSC. We showed explicitly that querying DeSigN using differentially expressed gene signatures could reveal potentially efficacious candidate drugs, as shown in the GSE4342 analyses. BIBW2992 (a newer generation of EGFR inhibitor currently approved for treating NSCLC patients who are refractory to gefitinib and erlotinib), for example, could potentially replace gefitinib, a first-generation EGFR tyrosine kinase inhibitors (TKI) that is increasingly becoming a non-viable solution as cancer cells of NSCLC patients treated with gefitinib inevitably develop resistance and relapse, with 8–10 months of median time to progression [26–28]

To date, many cases of successful drug repurposing studies have been reported, an exemplary study being that of methotrexate, a drug first developed for treating leukemia, and subsequently repurposed to treat a wide spectrum of cancers ranging from breast, ovarian, bladder to head and neck cancers [29, 30]. Here, we demonstrated the success of DeSigN in guiding the selection of bosutinib as a candidate drug against OSCC (a subset of HNSCC) cell lines. Emerging evidence supports the possible use of bosutinib for the treatment of HNSCC. First, the molecular target of bosutinib, Src has been reported to be a frequently altered gene in HNSCC and has been identified as a promising drug target [31]. Second, an analysis of gene expression data from 42 HNSCC cell lines also predicted that bosutinib has anti-tumour effect on HNSCC [25]. To the best of our knowledge this is the first time bosutinib was shown experimentally to have potency in OSCC cell lines.

While tools such as NFFinder, DMAP and FMCM that adopted the CMap concept make use of large public databases such as GEO, DrugMatrix, STITCH and HAPPI as their reference, DeSigN has its uniqueness whereby it explicitly capitalizes on the large panel of 707 human cancer cell lines in GDSC that have well-characterized gene expression and drug response data (Table 4). Specifically, DeSigN constructs drug-associated gene expression profile of resistant and sensitive cell lines from these 707 cell lines, whereas CMap associates response to a drug by constructing gene expression profiles of pre- and post-treatment conditions using only four cell lines. DeSigN utilizes the cumulative gene expression effect of many genes rather than one or a handful of genes, in this case global baseline DEGs. We believe through pan-cancer approach as suggested by The Cancer Genome Atlas (TCGA) Research Network, inherent genetic similarities between human cancer cell lines could result in the identification of relevant candidate drugs that have hitherto not been tested [32].

**Table 4:** Comparisons of tools that utilized Connectivity Map concept

Tools	Relationship feature	Reference database
DeSigN	Global baseline DEGs to drug response	GDSC
NFFinder	Transcriptomic data to drugs, diseases and experts	GEO, CMap and DrugMatrix
DMAP	Protein/gene to drug response	STITCH and HAPPI
FMCM	Pre- and post-treatment gene expression to drug response	CMap

The new leads derived from DeSigN are important for accelerating the discovery of new drugs for HNSCC treatment, which is currently limited to cetuximab, where this drug remains the only FDA-approved targeted therapy for advanced HNSCC [3]. Importantly, we would like to emphasize that all candidates with positive and significant Connectivity Score should be equally considered for validation instead of considering just the few top-ranked candidates, since factors such as cost of drug, ease of availability, method of administering, side effects and other factors, are important practical considerations in the clinical setting.

The current implementation of DeSigN uses differentially expressed genes as starting points to associate gene signatures with drug response phenotype. This input is not necessarily optimal, as genes that are involved in dysregulated pathways in the pathogenesis of cancer may not always have their expression substantially altered [33]. Since higher-order information such as network context and post-translational modification including reversible phosphorylation or acylation are not explicitly integrated in the current version, future improvements to DeSigN will focus on integrating these types of data.

For future work, we also intend to expand drug coverage in Version 1.0 of DeSigN by incorporating the gene expression and drug response data from Cancer Therapeutics Response Portal (CTRP) [34] and other large-scale pharmacogenomics studies. We anticipate that DeSigN will evolve as more cell line gene expression and drug response data become available.

## Conclusions

DeSigN provides proof-of-concept for the feasibility of using a computational approach to shortlist the most promising drug candidates for effective drug repurposing in cancer treatment. We expect that DeSigN will continue to evolve based on usage feedback from the community of cancer researchers, as well as improvements in methods for mining gene signatures that have strong network context.

## Additional files

Additional file 1: Table S1. List of sensitive and resistant cell lines for each of the 140 drugs in DeSigN. (XLS 88 kb)
Additional file 2: Table S2. Differentially expressed genes from ER-positive breast cancer versus normal (GSE42568). (XLS 204 kb)
Additional file 3: Table S3. Differentially expressed genes from GSE4342. (XLS 56 kb)
Additional file 4: Table S4. Differentially expressed genes from GSE16179. (XLS 266 kb)
Additional file 5: Table S5. Differentially expressed genes from GSE9633. (XLS 110 kb)
Additional file 6: Table S6. Differentially expressed genes from GSE35141. (XLS 45 kb)
Additional file 7: Table S7. Differentially expressed genes from OSCC cell lines. (XLS 38 kb)
Additional file 8: Figure S8. Mean IC_50_ of each cell line from MTT assay. The bars represent mean IC_50_ ± SE of at least three experiments. (TIF 705 kb)
Additional file 9: Table S9. Mean apoptotic cells relative to control (%). (XLS 40 kb)
Additional file 10: Table S10. Mean EdU^+^ cells relative to control (%). (XLS 47 kb)
Additional file 11: Figure S11. Bosutinib significantly inhibits the proliferation of OSCC cells in dose-dependent manner. OSCC cell lines were treated with bosutinib at 0.3–3 μM for 72 h and the effect of bosutinib on cell proliferation was determined by Click-iT cell proliferation assay. * denotes *p*-value < 0.05 relative to control untreated cells (0 μM). (TIF 785 kb)

## Abbreviations

CMap: Connectivity Map
DEG: Differentially expressed gene
DeSigN: Differentially Expressed Gene Signatures – Inhibitors
GDSC: Genomics of Drug Sensitivity in Cancer
NOK: Normal oral keratinocyte
OSCC: Oral squamous cell carcinoma

## References

[1] Hutchinson L, Kirk R (2011). High drug attrition rates--where are we going wrong?. Nat Rev Clin Oncol.

[2] Huang R, Southall N, Wang Y, Yasgar A, Shinn P, Jadhav A, Nguyen DT, Austin CP (2011). The NCGC pharmaceutical collection: a comprehensive resource of clinically approved drugs enabling repurposing and chemical genomics. Sci Transl Med.

[3] Vermorken JB, Mesia R, Rivera F, Remenar E, Kawecki A, Rottey S, Erfan J, Zabolotnyy D, Kienzer HR, Cupissol D (2008). Platinum-based chemotherapy plus cetuximab in head and neck cancer. N Engl J Med.

[4] Eberhard DA, Johnson BE, Amler LC, Goddard AD, Heldens SL, Herbst RS, Ince WL, Janne PA, Januario T, Johnson DH (2005). Mutations in the epidermal growth factor receptor and in KRAS are predictive and prognostic indicators in patients with non-small-cell lung cancer treated with chemotherapy alone and in combination with erlotinib. J Clin Oncol.

[5] Pao W, Wang TY, Riely GJ, Miller VA, Pan Q, Ladanyi M, Zakowski MF, Heelan RT, Kris MG, Varmus HE (2005). KRAS mutations and primary resistance of lung adenocarcinomas to gefitinib or erlotinib. PLoS Med.

[6] Chapman PB, Hauschild A, Robert C, Haanen JB, Ascierto P, Larkin J, Dummer R, Garbe C, Testori A, Maio M (2011). Improved survival with vemurafenib in melanoma with BRAF V600E mutation. N Engl J Med.

[7] Shoemaker RH (2006). The NCI60 human tumour cell line anticancer drug screen. Nat Rev Cancer.

[8] Chen JJ, Knudsen S, Mazin W, Dahlgaard J, Zhang B (2012). A 71-gene signature of TRAIL sensitivity in cancer cells. Mol Cancer Ther.

[9] Garnett MJ, Edelman EJ, Heidorn SJ, Greenman CD, Dastur A, Lau KW, Greninger P, Thompson IR, Luo X, Soares J (2012). Systematic identification of genomic markers of drug sensitivity in cancer cells. Nature.

[10] Barretina J, Caponigro G, Stransky N, Venkatesan K, Margolin AA, Kim S, Wilson CJ, Lehár J, Kryukov GV, Sonkin D (2012). The Cancer Cell Line Encyclopedia enables predictive modelling of anticancer drug sensitivity. Nature.

[11] Lamb J, Crawford ED, Peck D, Modell JW, Blat IC, Wrobel MJ, Lerner J, Brunet JP, Subramanian A, Ross KN (2006). The Connectivity Map: using gene-expression signatures to connect small molecules, genes, and disease. Science.

[12] Setoain J, Franch M, Martinez M, Tabas-Madrid D, Sorzano CO, Bakker A, Gonzalez-Couto E, Elvira J, Pascual-Montano A (2015). NFFinder: an online bioinformatics tool for searching similar transcriptomics experiments in the context of drug repositioning. Nucleic Acids Res.

[13] Huang H, Nguyen T, Ibrahim S, Shantharam S, Yue Z, Chen JY (2015). DMAP: a connectivity map database to enable identification of novel drug repositioning candidates. BMC Bioinformatics.

[14] Chung FH, Chiang YR, Tseng AL, Sung YC, Lu J, Huang MC, Ma N, Lee HC (2014). Functional Module Connectivity Map (FMCM): a framework for searching repurposed drug compounds for systems treatment of cancer and an application to colorectal adenocarcinoma. PLoS One.

[15] Subramanian A, Tamayo P, Mootha VK, Mukherjee S, Ebert BL, Gillette MA, Paulovich A, Pomeroy SL, Golub TR, Lander ES (2005). Gene set enrichment analysis: a knowledge-based approach for interpreting genome-wide expression profiles. Proc Natl Acad Sci U S A.

[16] Ritchie ME, Phipson B, Wu D, Hu Y, Law CW, Shi W, Smyth GK (2015). limma powers differential expression analyses for RNA-sequencing and microarray studies. Nucleic Acids Res.

[17] Xiao Y, Hsiao TH, Suresh U, Chen HI, Wu X, Wolf SE, Chen Y (2014). A novel significance score for gene selection and ranking. Bioinformatics.

[18] Clarke C, Madden SF, Doolan P, Aherne ST, Joyce H, O’Driscoll L, Gallagher WM, Hennessy BT, Moriarty M, Crown J (2013). Correlating transcriptional networks to breast cancer survival: a large-scale coexpression analysis. Carcinogenesis.

[19] Fadlullah MZ, Chiang IK, Dionne KR, Yee PS, Gan CP, Sam KK, Tiong KH, Ng AK, Martin D, Lim KP (2016). Genetically-defined novel oral squamous cell carcinoma cell lines for the development of molecular therapies. Oncotarget.

[20] Love MI, Huber W, Anders S (2014). Moderated estimation of fold change and dispersion for RNA-seq data with DESeq2. Genome Biol.

[21] Pau G, Fuchs F, Sklyar O, Boutros M, Huber W (2010). EBImage--an R package for image processing with applications to cellular phenotypes. Bioinformatics.

[22] El-Masry OS, Brown BL, Dobson PR (2012). Effects of activation of AMPK on human breast cancer cell lines with different genetic backgrounds. Oncol Lett.

[23] Gunzer K, Joly F, Ferrero JM, Gligorov J, de Mont-Serrat H, Uttenreuther-Fischer M, Pelling K, Wind S, Bousquet G, Misset JL (2016). A phase II study of afatinib, an irreversible ErbB family blocker, added to letrozole in patients with estrogen receptor-positive hormone-refractory metastatic breast cancer progressing on letrozole. Springerplus.

[24] Coldren CD, Helfrich BA, Witta SE, Sugita M, Lapadat R, Zeng C, Baron A, Franklin WA, Hirsch FR, Geraci MW (2006). Baseline gene expression predicts sensitivity to gefitinib in non-small cell lung cancer cell lines. Mol Cancer Res.

[25] Nichols AC, Black M, Yoo J, Pinto N, Fernandes A, Haibe-Kains B, Boutros PC, Barrett JW (2014). Exploiting high-throughput cell line drug screening studies to identify candidate therapeutic agents in head and neck cancer. BMC Pharmacol Toxicol..

[26] Maemondo M, Inoue A, Kobayashi K, Sugawara S, Oizumi S, Isobe H, Gemma A, Harada M, Yoshizawa H, Kinoshita I (2010). Gefitinib or chemotherapy for non-small-cell lung cancer with mutated EGFR. N Engl J Med.

[27] Sequist LV, Waltman BA, Dias-Santagata D, Digumarthy S, Turke AB, Fidias P, Bergethon K, Shaw AT, Gettinger S, Cosper AK (2011). Genotypic and histological evolution of lung cancers acquiring resistance to EGFR inhibitors. Sci Transl Med.

[28] Stinchcombe TE (2014). Recent advances in the treatment of non-small cell and small cell lung cancer. F1000Prime Rep.

[29] Vortherms AR, Dang HN, Doyle RP (2009). Anticancer conjugates and cocktails based on methotrexate and nucleoside synergism. Clin Med Oncol.

[30] Gupta SC, Sung B, Prasad S, Webb LJ, Aggarwal BB (2013). Cancer drug discovery by repurposing: teaching new tricks to old dogs. Trends Pharmacol Sci.

[31] Pickering CR, Zhang J, Yoo SY, Bengtsson L, Moorthy S, Neskey DM, Zhao M, Ortega Alves MV, Chang K, Drummond J (2013). Integrative genomic characterization of oral squamous cell carcinoma identifies frequent somatic drivers. Cancer Discov.

[32] Network Cancer Genome Atlas Research, Weinstein JN, Collisson EA, Mills GB, Shaw KR, Ozenberger BA, Ellrott K, Shmulevich I, Sander C, Stuart JM (2013). The Cancer Genome Atlas Pan-Cancer analysis project. Nat Genet.

[33] de la Fuente A (2010). From ‘differential expression’ to ‘differential networking’ - identification of dysfunctional regulatory networks in diseases. Trends Genet.

[34] Basu A, Bodycombe NE, Cheah JH, Price EV, Liu K, Schaefer GI, Ebright RY, Stewart ML, Ito D, Wang S (2013). An interactive resource to identify cancer genetic and lineage dependencies targeted by small molecules. Cell.

[35] Liu L, Greger J, Shi H, Liu Y, Greshock J, Annan R, Halsey W, Sathe GM, Martin AM, Gilmer TM (2009). Novel mechanism of lapatinib resistance in HER2-positive breast tumor cells: activation of AXL. Cancer Res.

[36] Wang XD, Reeves K, Luo FR, Xu LA, Lee F, Clark E, Huang F (2007). Identification of candidate predictive and surrogate molecular markers for dasatinib in prostate cancer: rationale for patient selection and efficacy monitoring. Genome Biol.

[37] Saiki Y, Yoshino Y, Fujimura H, Manabe T, Kudo Y, Shimada M, Mano N, Nakano T, Lee Y, Shimizu S (2012). DCK is frequently inactivated in acquired gemcitabine-resistant human cancer cells. Biochem Biophys Res Commun.

