# Supplementary figures and images for: DeSigN: connecting gene expression with therapeutics for drug repurposing and development
Source: BMC Genomics. 2017 Jan 25;18(Suppl 1):934. doi: 10.1186/s12864-016-3260-7 (PMC5310278; doi:10.1186/s12864-016-3260-7)

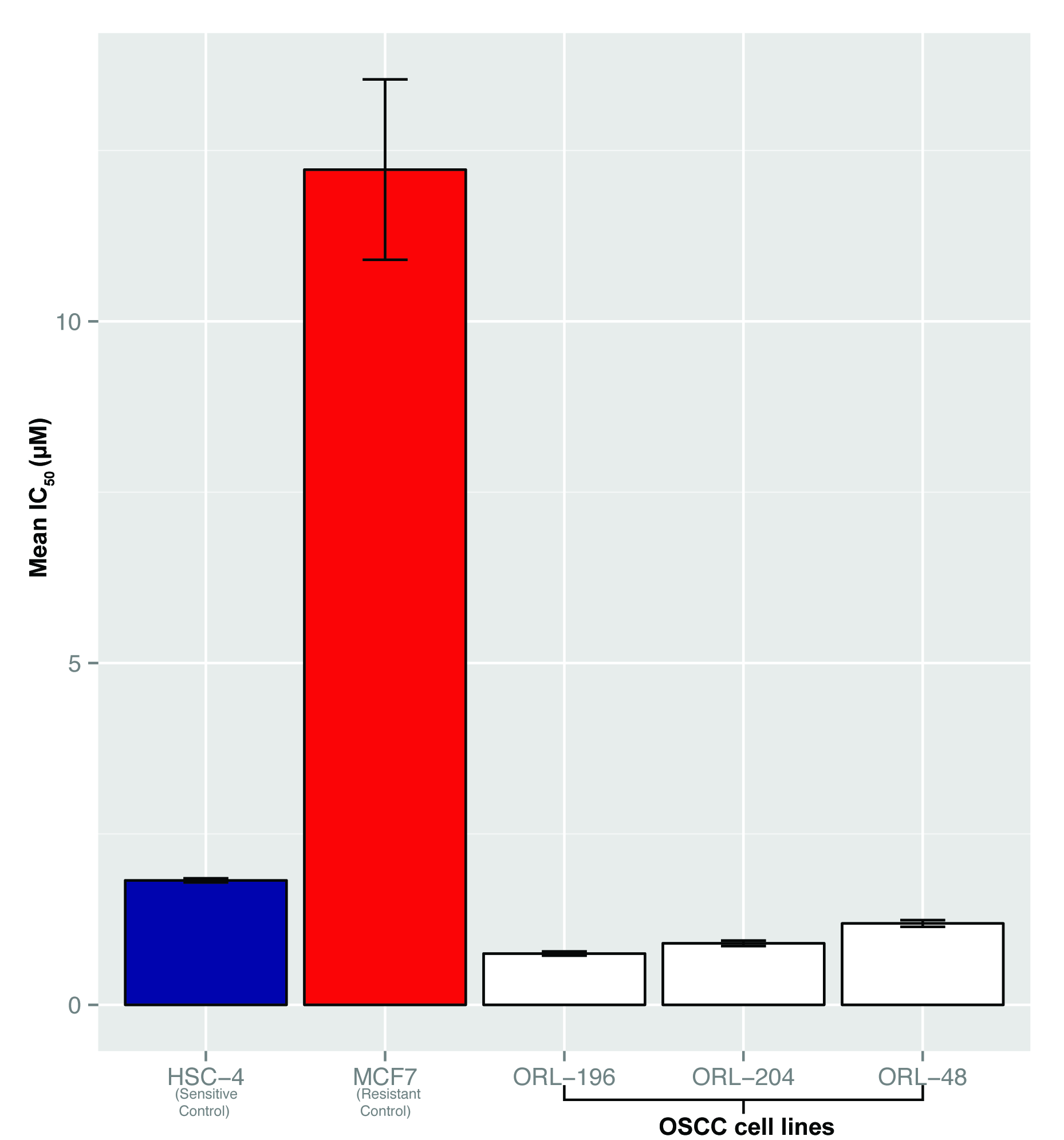

Supplement: Additional file 8: Figure S8. — Mean IC50 of each cell line from MTT assay. The bars represent mean IC50 ± SE of at least three experiments. (TIF 705 kb) [file 12864_2016_3260_MOESM8_ESM.tif]

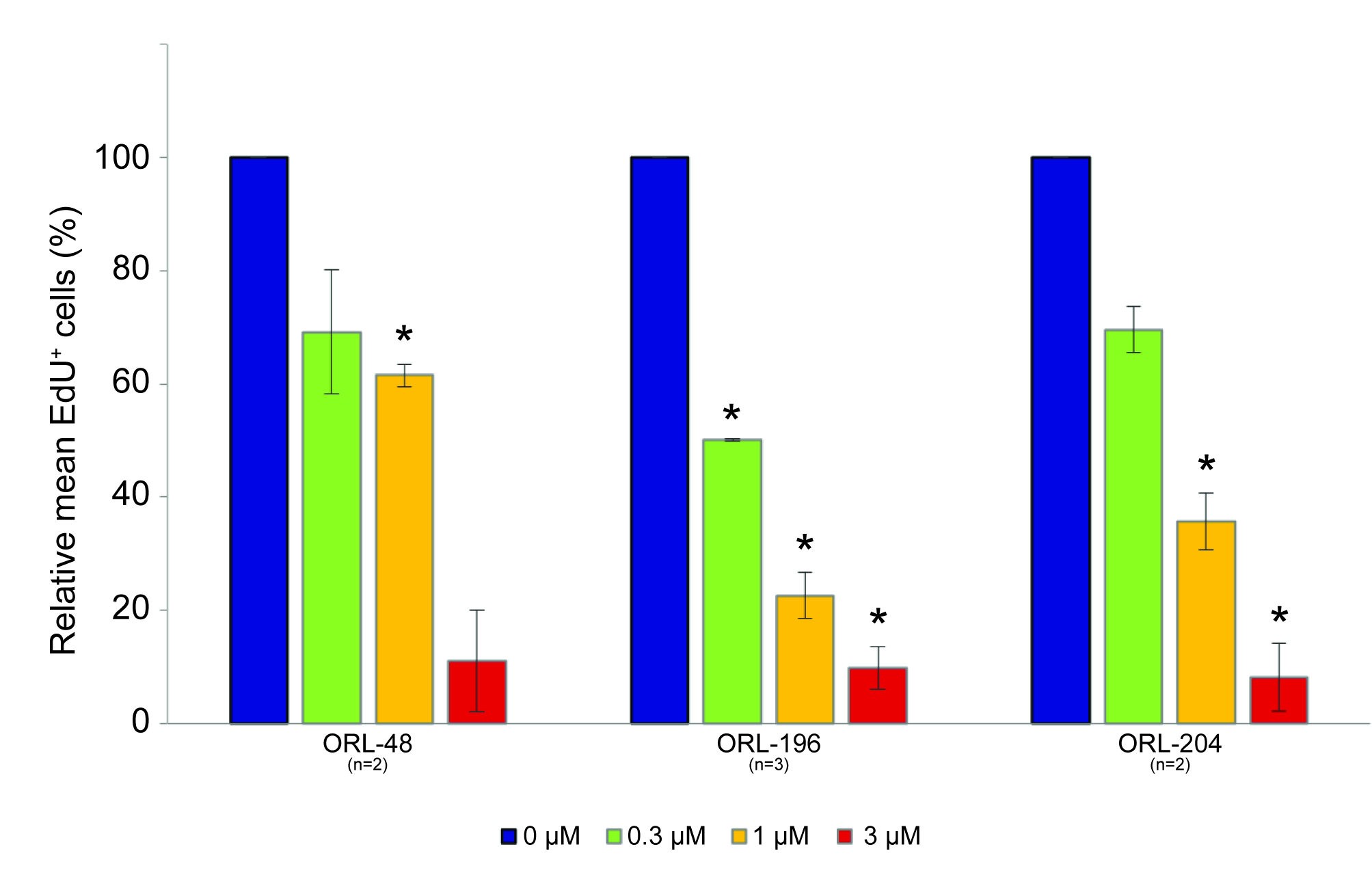

Supplement: Additional file 11: Figure S11. — Bosutinib significantly inhibits the proliferation of OSCC cells in dose-dependent manner. OSCC cell lines were treated with bosutinib at 0.3–3 μM for 72 h and the effect of bosutinib on cell proliferation was determined by Click-iT cell proliferation assay. * denotes p-value < 0.05 relative to control untreated cells (0 μM). (TIF 785 kb) [file 12864_2016_3260_MOESM11_ESM.tif]
